# Identification of homozygous haplotypes carrying putative recessive lethal mutations that compromise fertility traits in French Lacaune dairy sheep

**DOI:** 10.1186/s12711-021-00634-1

**Published:** 2021-05-01

**Authors:** Maxime Ben Braiek, Stéphane Fabre, Chris Hozé, Jean-Michel Astruc, Carole Moreno-Romieux

**Affiliations:** 1 GenPhySE, Université de Toulouse, INRAE, ENVT, 31326 Castanet-Tolosan, France; 2 Allice, 149 rue de Bercy, 75595 Paris, France; 3 Institut de l’Elevage, 24 chemin de Borde-Rouge, 31326 Castanet-Tolosan, France

## Abstract

**Background:**

Homozygous recessive deleterious mutations can cause embryo/fetal or neonatal lethality, or genetic defects that affect female fertility and animal welfare. In livestock populations under selection, the frequency of such lethal mutations may increase due to inbreeding, genetic drift, and/or the positive pleiotropic effects of heterozygous carriers on selected traits.

**Results:**

By scanning the genome of 19,102 Lacaune sheep using 50 k single nucleotide polymorphism (SNP) phased genotypes and pedigree data, we identified 11 Lacaune deficient homozygous haplotypes (LDHH1 to LDHH11) showing a highly significant deficit of homozygous animals ranging from 79 to 100%. These haplotypes located on chromosomes 3, 4, 13, 17 and 18, spanned regions from 1.2 to 3.0 Mb long with a frequency of heterozygous carriers between 3.7 and 12.1%. When we compared at-risk matings (between carrier rams and daughters of carrier rams) and safe matings, seven of the 11 haplotypes were associated with a significant alteration of two fertility traits, a reduced success of artificial insemination (LDHH1, 2, 8 and 9), and/or an increased stillbirth rate (LDHH3, 6, 8, 9, and 10). The 11 haplotypes were also tested for a putative selective advantage of heterozygous carrier rams based on their daughter yield deviation for six dairy traits (milk, fat and protein yields, fat and protein contents and lactation somatic cell score). LDHH1, 3, 4, 5, 7, 9 and 11 were associated with positive effects on at least one selected dairy trait, in particular milk yield. For each haplotype, the most probable candidate genes were identified based on their roles in lethality of mouse knock-out models and in mammalian genetic disorders.

**Conclusions:**

Based on a reverse genetic strategy, we identified at least 11 haplotypes with homozygous deficiency segregating in French Lacaune dairy sheep. This strategy represents a first tool to limit at-risk matings in the Lacaune dairy selection scheme. We assume that most of the identified LDHH are in strong linkage disequilibrium with a recessive lethal mutation that affects embryonic or juvenile survival in sheep but is yet to be identified.

**Supplementary Information:**

The online version contains supplementary material available at 10.1186/s12711-021-00634-1.

## Background

Most of the individuals in a population are likely to be heterozygous for several loss-of-function mutations. When these mutations are homozygous, they very often lead to early embryo death (embryonic lethal mutations), or to developmental defects that affect fetuses and subsequently young individuals with varying degrees of severity [1]. Advances in genomic approaches and whole-genome sequencing in humans or in species of agronomic interest have shown that an individual can carry about a hundred of these mutations [2, 3].

In livestock under selection, the effective size of populations (Ne), which is used as an indicator of genetic diversity, is limited (Ne ~ 100–300) compared with that of the human population (Ne ~ 10,000); and as a result, the number of reproducers is rather small, particularly given the widespread use of artificial insemination (AI) [2, 4]. Even if genetic diversity and inbreeding parameters are managed, selection programs provide a fairly favorable context for the emergence of homozygous individuals with genetic defects that increase in frequency with inbreeding and/or overuse of certain sires and can finally jeopardize fertility in the whole population [5]. This has been observed in cattle where about 1% of the embryos die due to their homozygosity at one of the 10 identified lethal embryonic mutations [1]. In addition, the frequency of recessive lethal alleles could also increase in a population if they are associated with heterozygous advantages due to positive pleiotropic effects on selected production traits such as milk production in dairy cattle [6, 7], although in the homozygous state they are responsible for embryonic losses. Identification of these causal mutations has become a major issue with the emergence of genetic defects with obvious consequences on animal welfare and also have major economic implications. Indeed, in France these disorders cause losses that range from 50 to 100 million euros per year in cattle populations when their impact on fertility (about 5% decrease), loss of calves, and veterinary procedures are included in the calculation [8].

In recent decades, several genomic tools have been developed to help improve fertility in dairy cattle [9]. Among these tools, two methods have enabled the identification and characterization of recessive genetic defects and lethal mutations that affect fertility. First, homozygosity-mapping is an efficient way to map genetic defects based on a case/control approach using only a few biological samples (e.g. DNA or tissues) from affected and non-affected live animals [10]. However, embryonic and fetal lethal mutations, which are more frequently associated with fertility, have not been identified using this approach due to the difficulty to obtain biological samples. These mutations are more efficiently detected by a reverse genetic screen approach using large sets of single nucleotide polymorphism (SNP) chip genotyped animals and fertility records, such as those provided by genomic selection. In cattle, the original works of VanRaden et al. [11] and Fritz et al. [12] were based on the identification of haplotypes for which homozygous carrier animals are absent or show a more significant homozygous haplotype deficiency (HHD) than expected. Their strategy used phased 50 k SNP genotypes from trios (offspring, sire, dam or maternal grand-sire), and the search for statistically significant HHD based on sliding windows of 20 to 100 SNPs. The underlying hypothesis is based on the linkage disequilibrium between these haplotypes and deleterious recessive mutations located nearby. This reverse genetic screen strategy has led to the identification of HHD regions that harbor 14 causal mutations in seven dairy cattle breeds. Among these, 11 HHD are associated with embryonic lethal mutations in Holstein [11–18], Jersey [19], Fleckvieh [20], Montbéliarde [12, 21], and Normande [22], and three are associated with juvenile mortality in Ayshire [23], Brown Swiss [24], and Fleckvieh [20]. With the recent increased use of genomic selection, the accumulation of genotyping data has enabled the identification of recessive lethal mutations by reverse genetic screening in other species such as pig and chicken [25, 26]. However, to date there are no such studies in sheep.

Compared to cattle, the management of genetic diversity in dairy sheep takes advantage of their more local selection and breed management and of the use of a wider range of rams to produce fresh semen during a short reproductive campaign (May to August) [27]. For example, the efficiency of the management of genetic diversity in Lacaune dairy sheep was explained by an effective population size of 336 [28]. However, since the implementation of genomic selection in 2015, the number of Lacaune rams that enter the AI program was reduced to balance the cost of genotyping [27]. Thus, the widespread use of a limited number of AI rams could favor the emergence of recessive alleles and possibly embryonic or fetal lethal mutations that affect fertility.

In order to discover such mutations, a reverse genetic screen method was applied to the large genome-wide SNP dataset available from a genomic selection program in Lacaune dairy sheep. The specific objectives of this study were to identify haplotypes with a deficit of homozygous animals, to test the hypothesis of a negative impact of these haplotypes on fertility traits in the case of at-risk matings, to test their putative pleiotropic effects on milk production traits, and to propose candidate genes that could harbor the causal mutations.

## Methods

### Animal and genotyping data

The genotyped dairy Lacaune animals [n = 19,102 born between 1996 and 2019 (see Additional file 1: Figure S1)] were obtained from the selection schemes of two breeding companies, OVITEST (Saint-Léon, France) and the *Confédération Générale de Roquefort* (Millau, France). Table 1 lists the details on all the animals used in the study (mainly rams) that were genotyped either on a medium-density (MD) SNP chip (Illumina Ovine SNP50 BeadChip, 54,241 SNPs, n = 12,600 genotyped animals between 1 and 12 months of age, born since 1996) or a low-density (LD) SNP chip (SheepLD v.1, 15,000 SNPs, n = 6502 genotyped animals between 1 and 5 months of age, born since 2017).

**Table 1:** Description of genotyped animals

Year of birth	≤ 2014	2015–2016	≥ 2017		Total
Background	Research programs	Genomic selection	Genomic selection		
Number of animals	6587 rams	3986 rams	7012 rams		19,102
	1517 ewes				
SNP chip	MD	MD	LD (n = 6502)	MD (n = 510)	LD (n = 6502)
					MD (n = 12,600)
Genotyping age (months)	> 12	1–5	1–5	8–12	

*MD* medium density (50 k), *LD* low density (15 k)

Both SNP chips (LD and MD) purchased from Illumina Inc. (San Diego, USA) were used to genotype the animals at Labogena (http://www.labogena.fr/) or Aveyron Labo (http://www.aveyron-labo.com/) genotyping facilities. Genotype data were obtained within the framework of different research programs before 2015, and subsequently from the ongoing Lacaune dairy sheep genomic selection program [29]. The pedigree of the genotyped animals was extracted from the official French livestock data system (*Systèmes Nationaux d’Information Génétique*, France Génétique Elevage, Paris, France).

### Genotype quality control, imputation and phasing

The quality control of each SNP was based on three criteria: (i) a call frequency higher than 97% (% of genotyped animals for each SNP), (ii) a minor allele frequency higher than 1%, and (iii) accordance with the Hardy–Weinberg equilibrium $({\text{P}} > 10^{ - 5} )$. LD to MD genotype imputation and phasing of all genotypes were implemented with the *FImpute v2.2* software [30]; the LD and MD chips had 11,342 common SNPs. The accuracy of LD to MD imputation of Lacaune genotypes was previously assessed and showed a concordance rate per animal of 99.05%, a concordance rate per SNP of 99.12%, and a squared Pearson’s correlation coefficient of 94.95% between imputed and observed SNP genotypes [31]. For subsequent identification of HHD, 38,696 SNPs on the 26 autosomal sheep chromosomes were available and mapped to the *Ovis aries* genome assembly Oar_v2.0 ([32], GigaDB, Oar_v2.0 coordinates are available at 10.5524/100023), the version used for the current genomic evaluation.

### Detection of homozygous haplotype deficiency

Based on phased MD genotype data, the ovine genome was scanned using a sliding window approach of 20 consecutive SNPs to identify HHD on the 26 sheep autosomes by comparing the observed and expected number of homozygous animals using the method developed by Fritz et al. [12] in French dairy cattle, and adapted to ovine data as described below.

For each window of 20 consecutive SNPs, the frequencies of all observed haplotypes were calculated from the maternal phase, which is associated with a greater diversity of haplotypes. Only haplotypes with a frequency higher than 1% were selected. The choice of a sliding window of 20 consecutive SNPs, representing approximatively 1.0 to 1.5 Mb (50 k SNP chip with an informative SNP every 60 kb, 3 Gb genome), and of a haplotype frequency higher than 1% was based on a previous simulation to estimate the frequency of recessive lethal mutations in breeding populations [2]. With an effective population size ranging from 100 to 500, as in Lacaune sheep (Ne = 336 [28]), the frequency of recessive lethal mutations is expected to range from 1 to 3%.

For each selected haplotype $k$, the number of observed homozygous animals ($N_{{{\text{Obs}}}} \left( k \right)$) was compared to the expected number of homozygous animals ($N_{{{\text{Exp}}}} \left( k \right)$). The number of expected homozygous animals $N_{Exp} \left( k \right)$ was estimated using the within-trio transmission probability with the formula described in Fritz et al. [12]. Two types of trios were considered, 38 progeny-sire-dam trios (transmission probability of haplotype $k$ is estimated based on the genotyped sire and dam) and 15,530 progeny-sire-maternal grandsire trios (transmission probability of haplotype $k$ is estimated based on the genotyped sire and maternal grandsire).

The probability of observing “$q$” homozygotes with an expectation “$lambda$” was estimated using the Poisson distribution and calculated with the *ppois* function $\left( {ppois\left( {q = N_{Obs} \left( k \right),lambda = N_{Exp} \left( k \right)} \right)} \right)$ in the RStudio software (Version 1.1.456), as previously described in Mesbab-Uddin et al. [22]. Each $k$ haplotype was assumed to be significantly deficient in homozygotes when the P-value was lower than 1.9 × 10^−4^. This threshold was obtained by a Bonferroni correction for multiple testing at a 5‰ level of significance assuming that the number of independent tests was equal to the number of chromosomes (n = 26). Among the significant haplotypes, only those with a severe deficiency that ranged from 75 to 100%, were retained as HHD $\left( {N_{Exp} \left( k \right) - N_{Obs} \left( k \right))/N_{Exp} \left( k \right) \ge 0.75} \right).$

When the significant HHD of 20 SNPs were consecutive (i.e., shifted from the previous one by a single SNP) and showed the same minimum number of homozygous animals, they were clustered together to define a larger haplotype (all these primary HHD are in total linkage disequilibrium with each other), which we refer to as ‘Lacaune deficient homozygous haplotype’ (LDHH) (see Additional file 2: Tables S1 and S2). The homozygous, heterozygous, and non-carrier status of each haplotype constituting an LDHH region was then determined for each animal in the studied population (n = 19,102).

Linkage disequilibrium was estimated between two LDHH regions on the same chromosome by the r^2^ coefficient measure that was introduced by Hill and Robertson [33]. For each LDHH region, a bi-allelic locus was defined as allele 1, i.e., the detected LDHH showing a deficit in homozygotes, and as allele 2, i.e., all other haplotypes identified in the same region. The coordinates of the SNPs included in each haplotype were obtained for the ovine genome assembly Oar_v2.0 and were repositioned on the genome assembly Oar_v3.1 [32] (available from GenBank, GCA_000298735.1) for further genetic analyses.

### Analysis of fertility and dairy production traits

#### Analysis of fertility traits

Trait records of Lacaune matings between 2006 and 2018 were obtained from the national database. We studied a first set of two fertility traits, i.e. artificial insemination success (AIS) and stillbirth rate (SBR). Among all the records, we focused only on matings between ewes with a genotyped sire and a genotyped ram, the sire and ram both having a known status at each LDHH (n = 1,155,835 matings). AIS was coded “1” for success and “0” for failure based on lambing date according to the gestation length starting from the day of AI (147 ± 5 days). SBR was determined only in the AI success group, and coded “1” if there was at least one stillbirth in the litter or “0” if all lambs were born alive (n = 804,577 matings). Four different types of mating are possible for each LDHH: (i) non-carrier ram × ewe from a non-carrier sire, (ii) non-carrier ram × ewe from a carrier sire, (iii) carrier ram × ewe from a non-carrier sire, and (iv) carrier ram × ewe from a carrier sire. Mating type (iv) was considered as at-risk mating, and the cluster of mating types (i), (ii) and (iii) were considered as safe mating. A logistic threshold binary model with a logit link function was used to compare AIS and SBR between at-risk and safe matings, using the GLIMMIX procedure in the SAS software (version 9.4; SAS Institute Inc., Cary, NC). The model used is ${\mathbf{y}} = {\mathbf{X}{\varvec{\upbeta}}} + {\mathbf{Z}\varvec{\upgamma }} + {\mathbf{e}}$, where ${\mathbf{y}}$ is a vector of “0” or “1” coding for AIS or SBR by considering the corresponding observations $Z$ (*Z* = {0,1} for AIS, *Z* = {0,1,2+} for SBR) and a threshold for each variable, so that ${\mathbf{y}} = 1 \;if\; Z \ge 1 \;or\; {\mathbf{y}} = 0 \;{\text{otherwise}}$; ${\mathbf{X}}$ is the incidence matrix of fixed effects; ${{\varvec{\upbeta}}}$ is a vector of fixed effects; ${\mathbf{Z}}$ is an incidence matrix of random effects; ${{\varvec{\upgamma}}}$ is a vector of random effects, and ${\mathbf{e}}$ is a vector of residual error effects. The fixed effects for AIS and SBR were mating type (safe or at-risk), month of AI (March to September), and lactation number (L1, L2, L3 and L4+). For SBR only, prolificacy of the ewe (1, 2, 3+ lambs/litter) was added as a fixed effect. For AIS and SBR, the random effect was interaction herd × year (n = 470 herds between 2006 and 2018). Traits were considered to differ significantly when the fixed effect mating type had a P-value lower than 6.3 × 10^–4^. This threshold was obtained by Bonferroni correction for multiple testing at a 1% level of significance by correcting the number of independent tests assumed to be the number of significant LDHH regions multiplied by the two independent fertility traits studied.

#### Analysis of milk parameters

Daughter yield deviations (DYD) for milk parameters from genotyped sires with known status at each LDHH were computed from genetic evaluations (GenEval, Jouy-en-Josas, France). The DYD corresponds to the average performance of the daughters of each sire, corrected for environmental effects and the average genetic value of the mothers [29]. The six parameters studied were milk yield (MY), fat (FC) and protein (PC) contents, fat (FY = MY × FC) and protein (PY = MY × PC) yields, and lactation somatic cell score (LSCS). LSCS corresponds to the average SCS per lactation, i.e. the log-transformation of test-day somatic cell count (SCC) defined by $SCS = log_{2} \left( {\frac{SCC}{{10000}}} \right) + 3$ [34–36]. To compare all the traits on the same scale, each DYD was divided by its genetic standard deviation and referred to as standardized DYD (sDYD). Only genotyped rams with records from at least 20 daughters were included in the analysis in order to obtain sufficiently accurate DYD values (n ~ 5400 rams). Each trait was tested by variance analysis comparing LDHH carrier and non-carrier rams using the GLM procedure in the SAS software (version 9.4; SAS Institute Inc., Cary, NC). The fixed effect model is ${\mathbf{y}} = {\mathbf{X }\varvec{\upbeta}} + {\mathbf{e}}$, where ${\mathbf{y}}$ is a vector of sDYD data for each trait; ${\mathbf{X}}$ is an incidence matrix of fixed effects; ${{\varvec{\upbeta}}}$ is a vector of the fixed effects and ${\mathbf{e}}$ is a vector of residual error effects. The fixed effects are the genetic status (carrier, non-carrier) and year of birth (2000 to 2016) to correct for annual genetic gain. Traits differ significantly between carrier and non-carrier rams when the effect of the genetic status is significant, i.e. with a P-value lower than 3.1 × 10^–4^. This threshold was obtained by Bonferroni correction for multiple testing at a 1% level of significance by correcting the number of independent tests assumed to be the number of significant LDHH regions multiplied by the four studied traits (MY, FC, PC and LSCS).

### Identification of positional and functional candidate genes

The coordinates of each LDHH region were obtained from the ovine genome assembly Oar_v3.1. and extended 1 Mb upstream and 1 Mb downstream to obtain gene annotations from the Ensembl release 99 using the Biomart tool [37] (accessed on 20/03/2020; see Additional file 3: Table S3). Annotations and genome organization within these regions were found to be the same as those of the most recent ovine genome assembly Rambouillet v1.0 (NCBI *Ovis aries* Annotation Release 103, 2019-02-06, GCF_002742125.1).

Gene information (gene description, biological process, and molecular function) was extracted from several databases (NCBI Entrez: https://www.ncbi.nlm.nih.gov/Web/Search/entrezfs.html; MGI: www.informatics.jax.org; IMPC: https://www.mousephenotype.org). The list of identified genes was then sorted to identify the most relevant candidate genes according to (i) their known implication in lethal phenotypes in knockout/loss-of-function mouse models based on viability information in the IMPC database and mortality/aging (embryonic, prenatal, perinatal, neonatal, postnatal, preweaning, premature death) information in the MGI database, and (ii) their association with abortion/death/autosomal recessive disorders from Online Mendelian Inheritance in Man (OMIM) (https://omim.org) and OMIA: Online Mendelian Inheritance in Animal (https://omia.org).

## Results

### Identification of HHD in Lacaune dairy sheep

By screening the genome of 16,346 animals (belonging to trios) from real/imputed 50 k genotyping data, we detected 266 highly significant HHD of 20 consecutive SNPs, each with a frequency higher than 1% and a deficit of homozygous animals higher than 75%. The location of these haplotypes along the ovine genome is shown as a Manhattan plot (see Additional file 4: Figure S2). As explained in “Methods” section, when significant HHD of 20 SNPs were consecutive (shifted by a single SNP) and showed the same minimum number of homozygous animals, they were clustered to define 11 larger haplotypes containing 23 to 48 SNPs and named LDHH (Table 2).

**Table 2:** List of Lacaune deficient homozygous haplotypes

Haplotype	OAR	Number of markers^a^	Position^b^ (Mb)	Carrier frequency^c^ (%)	Number of homozygotes			
					Exp^d^	Obs^e^	Deficit (%)	Poisson P-value
LDHH1	4	46/42	43.4–46.3	6.7	21	0	100	7.6 × 10^−10^
LDHH2	13	28/26	44.8–46.8	6.2	17	0	100	4.1 × 10^−8^
LDHH3	3	48/39	32.0–34.9	4.3	10	0	100	7.0 × 10^−5^
LDHH4	3	24/19	132.4–133.9	3.8	10	0	100	5.5 × 10^−5^
LDHH5	3	29/23	131.1–132.7	3.7	9	0	100	1.9 × 10^−4^
LDHH6	3	27/21	136.2–137.4	12.1	72	3	96	3.5 × 10^−27^
LDHH7	17	29/27	0.0–1.6	4.7	12	1	92	9.9 × 10^−5^
LDHH8	18	23/20	25.7–27.5	5.5	14	2	86	9.4 × 10^−5^
LDHH9	18	37/33	31.3–33.5	4.4	13	2	85	2.2 × 10^−4^
LDHH10	18	26/23	33.0–34.6	5.7	17	3	82	4.1 × 10^−5^
LDHH11	3	28/28	128.9–131.1	7.2	19	4	79	3.8 × 10^−5^

^a^Number of LDHH markers refers to ovine genome reference assembly Oar_v2.0/Oar_v3.1 and listed in Additional file 2: Table S3

^b^Position on ovine genome assembly Oar_v3.1

^c^Frequency of carriers in the entire genotyping population (n = 19,102)

^d^Expected

^e^Observed

Among these haplotypes, five LDHH presented a complete deficit of observed homozygous animals (LDHH1 to 5) and six LDHH presented a partial deficit ranging from 79 to 96% of the expected number of homozygous animals (LDHH6 to 11). The length of the identified haplotypes ranged from 1.2 to 3.0 Mb on the ovine genome v3.1. LDHH3, 4, 5, 6 and 11 are located on *Ovis aries* (OAR) chromosome 3. Only LDHH4 (24 SNPs) and LDHH5 (29 SNPs) are in high linkage disequilibrium (71%). Although they share nine SNPs at their ends, LDHH4 and LDHH5 were not originally clustered together since some consecutive 20-SNP HHD between the two LDHH did not follow our clustering rule (they were neither significant nor had the same minimum number of homozygous animals). LDHH8 is located on OAR18, 3.7 Mb from LDHH9 and 5.4 Mb from LDHH10, and shows a moderate linkage disequilibrium of 55% and 40% with these two haplotypes, respectively. Likewise, LDHH9 (37 SNPs) and LDHH10 (26 SNPs) are in high linkage disequilibrium (72%), share 11 SNPs, but were not clustered together for the same reason as LDHH4 and 5. Other LDHH are located on OAR4 (LDHH1), OAR13 (LDHH2) and OAR17 (LDHH7). Consequently, the 11 LDHH identified are most probably associated with only eight independent causal mutations in the eight following genomic regions on OAR3 (LDHH3, 32.0–34.9 Mb; LDHH11, 128.9–131.1 Mb; LDHH4–5, 131.1–133.9 Mb and LDHH6, 136.2–137.4 Mb), OAR4 (LDHH1, 43.4–46.3 Mb), OAR13 (LDHH2, 44.8–46.8 Mb), OAR17 (LDHH7, 0–1.6 Mb) and OAR18 (LDHH8–9–10, 25.7–34.6 Mb). When calculated based on the whole genotyped population, the frequency of carriers ranged from 3.7 to 6.7% for LDHH with a total deficit of homozygotes, and from 4.4 to 12.1% for LDHH with a partial deficit.

### Impact of LDHH on the success rate of AI and on stillbirth rate

**Fig. 1:**
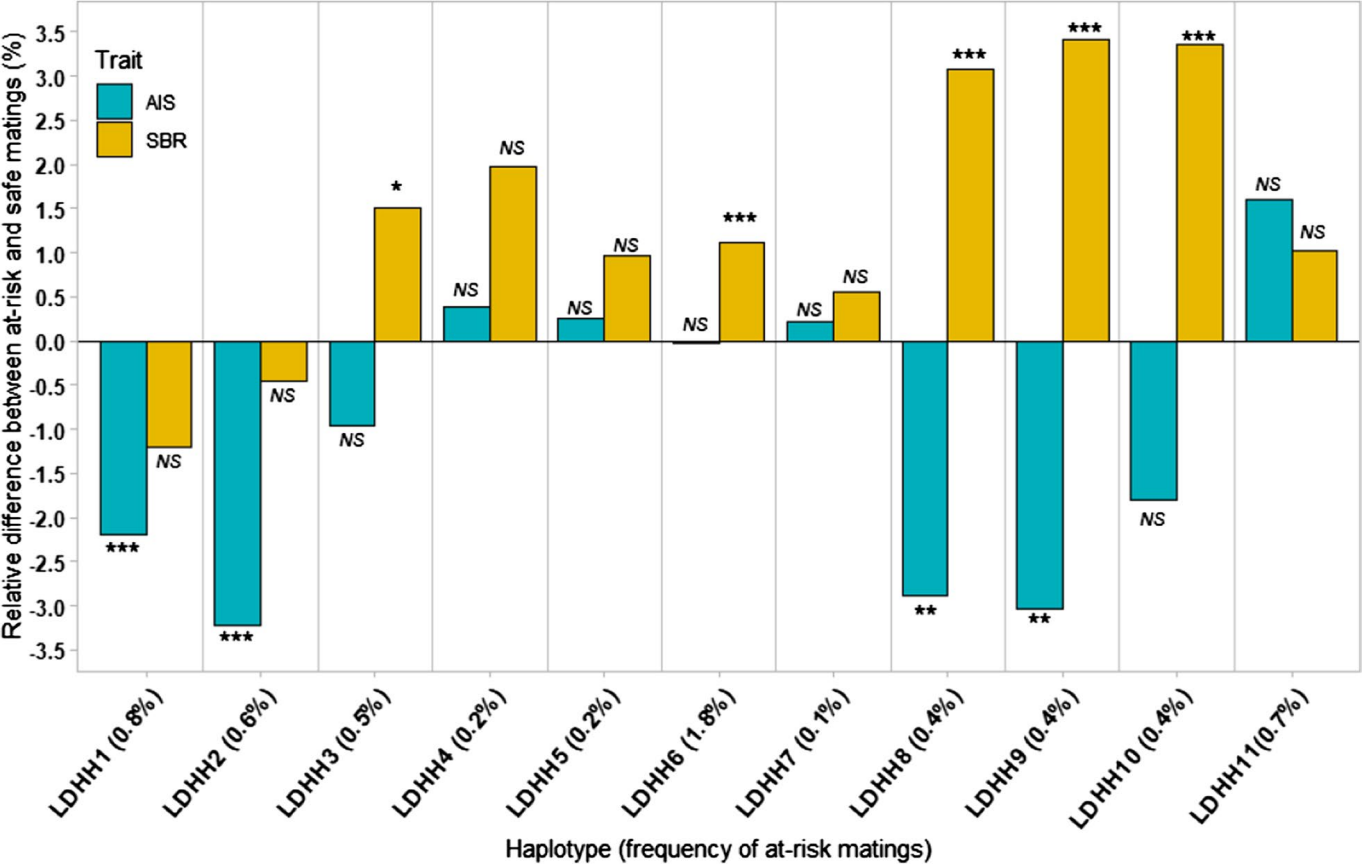
Effects of LDHH on the success rate of artificial insemination and on the stillbirth rate in at-risk matings compared to safe matings. *AIS* artificial insemination success, *SBR* stillbirth rate. For each LDHH, the frequency of at-risk mating is shown in parentheses. Significant effects are indicated by the corrected P-value for multiple tests with a threshold set at α = 1%: *P < 6.3 × 10^−4^; **P < 6.3 × 10^−5^ and ***P < 6.3 × 10^−6^

To check for a putative effect of the 11 LDHH on embryonic, fetal and/or perinatal lethality in the dairy Lacaune population, two fertility-associated traits that could reflect the consequences of these mutations were analyzed: AI success (AIS: 1,155,835 matings) and stillbirth rate (SBR: 804,577 matings) (Fig. 1).

AIS could be a good proxy for embryonic losses during the first weeks after AI. In our study population, the average AIS was 69.6%. Interestingly, at-risk matings for four LDHH (1, 2, 8 and 9) had a significant negative effect on AIS leading to a decreased success rate of 2.2% for LDHH1 (P = 3.2×10^−6^) up to 3.2% for LDHH2 (P = 2.6×10^−9^).

In parallel, evaluating the stillbirth rate could be a useful way to identify mutations that cause perinatal lethality. In our study population, 5.1% of litters included at least one stillbirth. Five significant LDHH (3, 6, 8, 9 and 10) were associated with a 1.1% increase in SBR for LDHH6 (P = 5.9×10^−7^) up to 3.4% for LDHH9 (P = 8.5×10^−12^) in at-risk compared to safe matings.

### Pleiotropic effects of LDHH on milk production traits

**Fig. 2:**
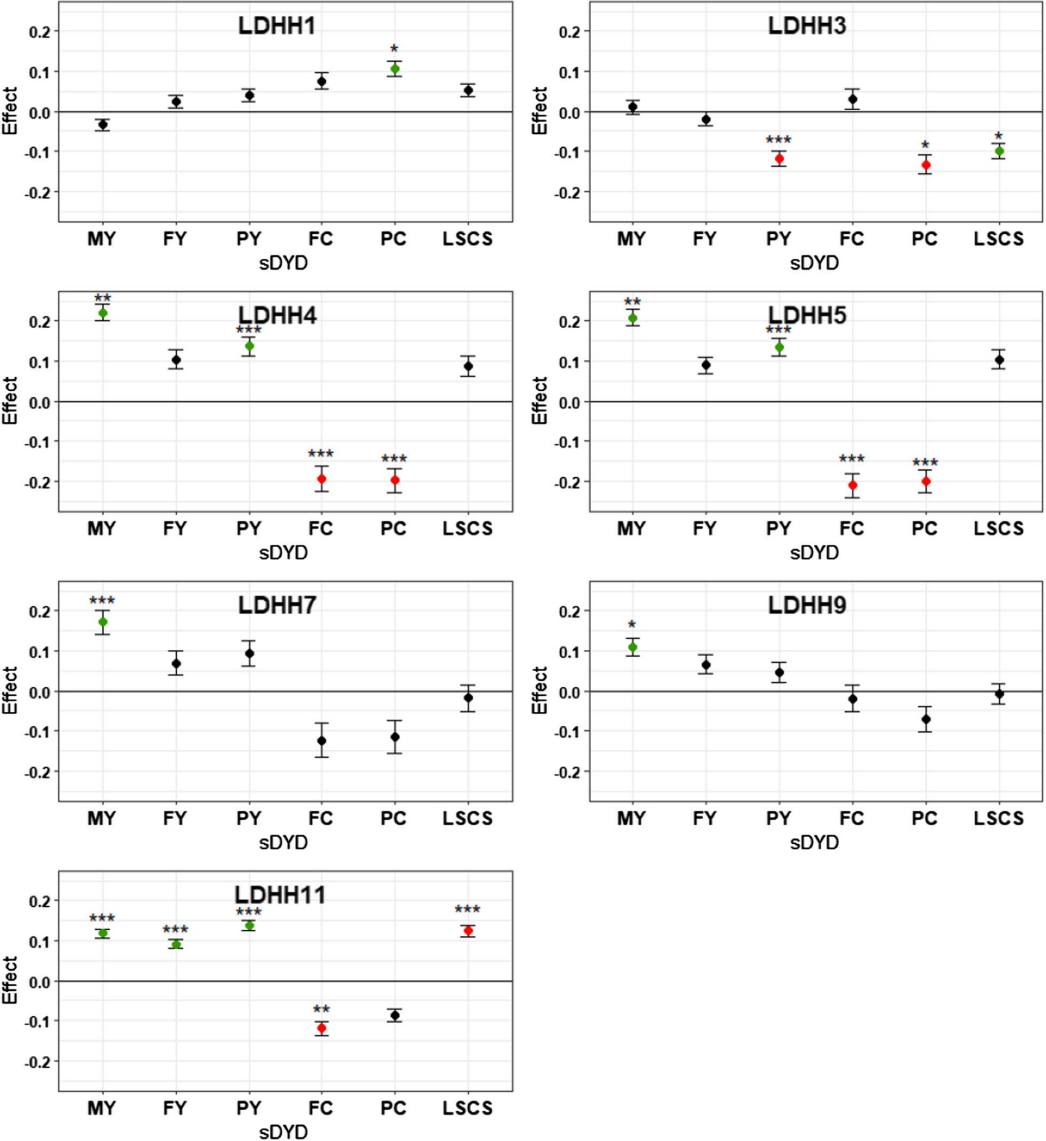
sDYD relative difference between heterozygous and non-carrier rams for 6 selected traits. *MY* milk yield, *FY* fat yield, *PY* protein yield, *FC* fat content, *PC* protein content, *LSCS* Lactation somatic cell score, *sDYD* standardized daughter yield deviation (DYD divided by genetic standard deviation). Significant effects are indicated by the corrected P-value for multiple tests with a threshold set at α = 1%: *P < 3.1 × 10^−4^; **P < 3.1 × 10^−5^ and ***P < 3.1 × 10^−6^. Error bars indicate standard errors. Significant favorable effects of heterozygous are in green and significant unfavorable effects are in red

For the 11 LDHH regions detected in dairy Lacaune, we tested whether a putative selective advantage existed at the heterozygous state for six dairy traits routinely included in genomic evaluations. sDYD of five milk production traits (milk, fat and protein yields, and fat and protein contents) and lactation somatic cell score (a proxy for mastitis) were compared between carrier and non-carrier rams of each LDHH (n ~ 5400 genotyped rams with DYD). Figure 2 shows the relative differences in sDYD calculated between heterozygous and non-carrier rams. For all milk production traits, positive values indicate an improvement of the selected traits, whereas for the lactation somatic cell score, a positive value indicates a deterioration of udder health in the progeny of heterozygous rams.

Among the 11 haplotypes, seven had a significant effect on sDYD for at least one trait. Daughters of LDHH1 carrier rams had a higher protein content (sDYD + 0.11), whereas those of LDHH3 carriers had a significantly lower protein yield (sDYD − 0.12) and protein content (sDYD − 0.13). Moreover, LDHH3 was significantly associated with a lower somatic cell score in milk (sDYD − 0.10). Both LDHH4 and LDHH5 carriers showed a significant increase in milk production (sDYD + 0.22 and + 0.21, respectively) and in protein yield (sDYD + 0.14 and + 0.13, respectively) but had negative effects on fat (sDYD − 0.19 and − 0.21, respectively) and protein content (sDYD − 0.20 in both). LDHH7 and LDHH9 carriers showed a higher sDYD for milk production with an increase of + 0.17 and + 0.11, respectively. LDHH11 was also associated with a + 0.12 increase in sDYD for milk production, and a wider effect spectrum, a positive effect on fat and protein yields sDYD (with + 0.09 and + 0.14, respectively), but a negative effect on LSCS (+ 0.12) and fat content sDYD (− 0.12).

### Candidate genes from LDHH regions

Among the 340 protein coding genes identified in the 11 LDHH regions extended by 1 Mb on each side (see Additional file 3: Table S3), 59 are associated with lethality when invalidated in mice (all homozygous mutant alleles lead to lethality, complete penetrance) and 40 show sub-viable phenotypes (higher deficit of homozygous mutant animals than expected according to Mendelian inheritance, incomplete penetrance). Among these genes, several are listed in the OMIM and OMIA databases as being associated with mammalian autosomal recessive disorders (Table 3). Most of these 99 genes are involved in nucleic acid synthesis/DNA replication/transcription (*PCNA*, *CAD*, *HOXC13*, and *ARNT2*), or encode structural or signaling proteins (*MAGI2*, *RELN*, *PRNP*, *FERMT1*, *IFT172*, *CEP83*, *KRT8*, *WNT1*, *CCDC65*, and *TLL1*) or are involved in basal metabolic processes (*PMPCB*, *IDI1*, *POMC*, *HADH-A/B*, *EIF2B4*, *SCN8A*, *PFKM*, *FAH*, *MPI*, *CYP1A2*, *STRA6*, and *CRADD)*. Knock-out mouse models of these genes have mainly revealed defects that affect offspring soon after birth (perinatal to weaning stages) and jeopardize the survival of the young.

**Table 3:** Most probable candidate genes affecting viability in mouse knockout models and associated with mammalian autosomal recessive disorders

Haplotype	Gene name^a^	Gene description	Mammalian recessive disorder^b^
LDHH1	*MAGI2*^†^	Membrane associated guanylate kinase, WW and PDZ domain containing 2	Nephrotic syndrome
	*PMPCB*^†^	Peptidase, mitochondrial processing beta subunit	Multiple mitochondrial dysfunctions syndrome
	*RELN*^†*^	Reelin	Lissencephaly and cerebellar hypoplasia
LDHH2	*IDI1*^†^	Isopentenyl-diphosphate delta isomerase 1	Zellweger syndrome and neonatal adrenoleukodystrophy
	*PRNP*^†^	Major prion protein	Spongiform encephalopathy
	*PCNA*^†^*	Proliferating cell nuclear antigen	Ataxia-telangiectasia-like disorder
	*FERMT1*^†^	Fermitin family member 1	Kindler syndrome
LDHH3	POMC*	Proopiomelanocortin	Obesity, adrenal insufficiency
	*HADHA*^†^, *HADHB*^†^	Hydroxyacyl-CoA dehydrogenase trifunctional multienzyme complex subunit alpha/beta	Long-chain 3-hydroxyacyl-CoA dehydrogenase deficiency, trifunctional protein deficiency with myopathy and neuropathy
	*CAD*^†^	Carbamoyl-phosphate synthetase 2, aspartate transcarbamylase, and dihydroorotase	Abortion; epileptic encephalopathy, early infantile
	*EIF2B4*^†^	Eukaryotic translation initiation factor 2B subunit delta	Leukoencephalopathy, ovarioleukodystrophy
	*IFT172*^†^	Intraflagellar transport 172	Retinitis pigmentosa, short-rib thoracic dysplasia ± polydactyly
LDHH4–5	HOXC13*	Homeobox C13	Ectodermal dysplasia
	*KRT8*^†^*	Keratin 8	Cryptogenic cirrhosis
	*SCN8A*^†^*	Sodium voltage-gated channel alpha subunit 8	Spinocerebellar ataxia
LDHH6	*WNT1*^†^*	Wnt family member 1	Osteogenesis imperfecta, type XV
	CCDC65*	Coiled-coil domain containing 65	Primary ciliary dyskinesia
	PFKM*	Phosphofructokinase, muscle	Glycogen storage disease VII
LDHH7	TLL1*	Tolloid like 1	Heart malformation
LDHH8-9–10	*FAH*^†^	Fumarylacetoacetate hydrolase	Tyrosinemia
	*ARNT2*^†^*	Aryl hydrocarbon receptor nuclear translocator 2	Webb-Dattani syndrome
	*MPI*^†^	Mannose phosphate isomerase	Congenital disorder of glycosylation, type Ib
	CYP1A2*	Cytochrome P450 family 1 subfamily A member 2	Metabolizer of a cognitive enhancer
	*STRA6*^†^	Stimulated by retinoic acid 6	Microphthalmia
LDHH11	*CRADD*^†^	CASP2 and RIPK1 domain containing adaptor with death domain	Mental retardation, with variant lissencephaly
	*CEP83*^†^	Centrosomal protein 83	Nephronophthisis

^a^Homozygous lethal^†^ (complete penetrance) and homozygous sub-viable* (incomplete penetrance) genes affecting developmental stages reported in knockout databases (IMPC and/or MGI)

^b^Mammalian autosomal recessive disorders reported in OMIM and/or OMIA databases

## Discussion

We identified eight independent genomic regions (named LDHH) in Lacaune dairy sheep showing complete or partial deficit of homozygous haplotypes, which suggests that several independent recessive deleterious mutations segregate in this population. To successfully identify HHD in dairy sheep, we used the same criteria as those used in dairy cattle, i.e. sliding windows of 20 consecutive SNPs and considered significant haplotypes with a frequency higher than 1% [12]. Using about 20,000 related animals genotyped with the 50 k SNP chip, the number of HHD identified and the carrier frequencies (between 4 and 12%) in dairy sheep are in line with the estimations reported in cattle [17]. Detection of HHD segregating at a lower frequency would have required more animals, but this will be possible in the near future thanks to the ongoing accumulation of genotyping data. Even if the length of these haplotypes depends on population structure, genome size, or the density of the SNP chip used, the length (from 1.2 to 3.0 Mb) of the LDHH identified here is in line with previously reported observations in other livestock with haplotypes spanning regions of less than 5 Mb [1]. One notable difference between our study on sheep and the literature on cattle is that less than 1% of the genotyped Lacaune animals have their two parents genotyped. Indeed, in Holstein and Normande cattle for which HHD were identified, this number reaches 30%. However, in sheep, dams are not routinely genotyped for genomic selection programs, which reduces the HHD detection power of our analysis since, in the trios included here, most of the dam genotypes are estimated by the status of the maternal grandsire and the frequency of the haplotype in the population.

In our population, we estimated that the frequencies of LDHH carriers ranged from 3.7 to 12.1%, which is in line with those of previous studies on cattle and pig (recently reviewed in Georges et al. [1]). Considering that these haplotypes are associated with deleterious mutations in the homozygous state, such frequencies could be considered as being quite high compared to the estimated frequency in humans, which is less than 1% [2]. In livestock populations with a small effective size (Ne = 336 in dairy Lacaune [28]), allele frequencies fluctuate randomly from one generation to the next due to small sampling of reproducers. Thus, the frequency of a deleterious recessive mutation can increase sharply by genetic drift from an initial value close to 0 up to several percent, as we observed in Lacaune (from 2 to 6%), due to the spread of very influential carriers and their progeny [5]. The Lacaune sheep population has a complex history with the creation of two lines, one for meat and one for dairy purposes, and four independent selection schemes. Based on this particular population structure, further simulation studies would be useful to estimate the effect of drift on the frequency of LDHH carriers. Apart from genetic drift, maintaining or increasing the frequency of deleterious alleles in a livestock population can be accomplished by balancing selection when deleterious alleles provide a heterozygous advantage on selected traits [5]. Several examples of homozygous deleterious mutations have been reported with a heterozygous advantage in livestock [38], such as the 660-kb deletion (spanning four genes) identified in Nordic Red cattle that leads to higher milk production in heterozygous carriers [7], the 2-pb deletion in the *MRC2* gene leading to the Crooked Tail syndrome in Belgian Blue cattle, but enhances muscular development in heterozygous carriers [39], or the 212-kb deletion affecting the *BBS9* and *BMPER* genes associated with higher growth rates in pig [40]. In the current study, seven of the 11 LDHH (i.e. six of the eight independent regions) are associated with positive effects on at least one milk trait (milk, protein and fat yields, protein and fat contents and LSCS) in the heterozygous state. The observed difference between carrier and non-carrier rams corresponds mainly to 1 or 2 years of genetic gain for the given traits [34]. For example, the observed difference in milk yield corresponds to + 4.0 L up to + 8.1 L for LDHH9 and LDHH4 carriers, respectively. These results are relatively similar to the effect observed for lethal mutations detected in US dairy cattle [6]. Surprisingly, LDHH6 which is the most frequent haplotype in our study (carrier frequency of 12.1%) had no pleiotropic effect on milk traits, which was also the case of the less frequent LDHH2, LDHH8 and LDHH10. Apart from the hypotheses of genetic drift or association with selected traits, the high frequency observed for LDHH6 could be explained by balancing selection of other traits that are obviously not implemented in the selection scheme, such as morphological phenotypes that match breed criteria (stature, fleece type and color, hornless).

Based on linkage disequilibrium, the 11 LDHH identified delimit only eight independent genomic regions probably associated with eight causal mutations. Within these regions, we searched for candidate genes that may be affected by these mutations in accordance with the characteristics of the genotyped population, the deficit of homozygous animals and the observed impact on AIS and SBR (assuming lethality from the embryonic to the juvenile stage). We reviewed mouse knockout models and report genetic disorders that are listed in human and animal databases (Table 3). Knowledge based on bovine studies shows that recessive lethal alleles are caused by loss-of-function mutations (stop-gain, frameshift, missense, splice site and deletion) affecting genes mainly involved in cell division, DNA replication, transcription, RNA processing, or coding for structural proteins or in essential metabolic processes [2, 41]. Based on AIS and SBR analyses, we were able to classify LDHH into four patterns, each with a hypothesized associated effect. The first pattern groups LDHH1 on OAR4 and LDHH2 regions on OAR13. Both haplotypes have a significant impact on AIS that we associate with early embryonic loss. Accordingly, we found that *PMPCB* and *PCNA* are strong candidate genes in the LDHH1 and LDHH2 regions, respectively. Indeed, homozygous invalidation of both genes in mouse causes embryonic lethality around the implantation stage [MGI:1920328, MGI:97503]. The second pattern corresponds to LDHH that have an impact on both AIS and SBR and that we associate with embryo/fetal loss throughout the gestation period, i.e. LDHH8, 9 and 10 on OAR18. In this case, we suspect a unique lethal mutation that affects one of the four following candidate genes *FAH*, *ARNT2*, *MPI* and *STRA6*, which are reported to be homozygous lethal in knock-out mouse models (*Fah* [MGI:95482], *Arnt2* [MGI:107188], *Mpi* [MGI:97075] [42] and *Stra6* [MGI:107742]). The third pattern groups LDHH3 and LDHH6 on OAR3 and affects only SBR. We assume that these LDHH harbor lethal mutations with effects that occur at the end of gestation and/or very soon after birth. Within the LDHH3 region in complete homozygous deficiency, the *CAD* gene is the most obvious candidate. Indeed, invalidation of the *Cad* gene in mouse causes preweaning lethality with complete penetrance [MGI:1916969], and a missense mutation in *CAD* (g.72399397T>C; p.Tyr452Cys) associated with the NH7 homozygous deficient haplotype [OMIA 002201-9913] causes late abortion during the last months of gestation in Normande cattle [22]. In the LDHH6 region, three genes *Wnt1*, *Ccdc65*, and *Pfkm,* are associated with perinatal, neonatal or preweaning lethality in mouse knock-out models [MGI:98953, MGI:2146001, MGI:97548]. Among these, a mutation in *CCDC65* encoding a nexin-dynein regulatory complex, is reported to cause “ciliary dyskinesia, primary 27” [CILD27, OMIM #615504], which is a neonatal respiratory distress syndrome in humans [43]. Interestingly, a respiratory syndrome is also reported for the homozygous deficient Braunvieh haplotype 2 (BH2) [OMIA 001939-9913] associated with perinatal and juvenile mortality in cattle due to a mutation in *TUBD1 (tubulin delta 1)* that disorganizes the microtubules in airway cilia [24]. Finally, the fourth pattern groups LDHH4–5, LDHH7 and LDHH11 for which we failed to produce evidence for an alteration of fertility traits when comparing at-risk and safe matings. This could be due to the relatively low frequency of at-risk matings in our dataset for LDHH4–5 (0.2%) and LDHH7 (0.1%), which made it impossible to reach statistical significance. Alternatively, these haplotypes could also host mutations that affect lamb survival in a later growth period, as observed for heart malformation in mouse [44] and in humans (“atrial septal defect 6” [ASD6, OMIM #613087]; [45]) associated with the *TLL1* gene located in the LDHH7 region. The last hypothesis for these LDHH in the fourth pattern concerns an effect related to morphological defects or breed standards that is possibly counter-selected in selection schemes.

Particular attention should also be focused on OAR3 and the region spanned by LDHH4–5 and LDHH11. These haplotypes are located in the region of the p.R96C mutation (OAR3, g.129722200C>T, genome assembly v3.1) that has been previously detected in the *suppressor of cytokine signaling 2* (*SOCS2*) gene in Lacaune dairy sheep and is associated with sensitivity to mastitis [46]. Interestingly, in our study, these LDHH, and particularly LDHH11 that includes *SOCS2*, had positive effects on milk production, on fat and protein yields, but negative effects on LSCS and fat content, as already observed for the C variant of the p.R96C mutation. Since 2017, genotypes for OAR3:129722200C>T are available for all animals genotyped on the LD chip when candidate rams are selected to enter the breeding center. Although LDHH4 and LDHH5 are not in linkage disequilibrium with LDHH11, all three are associated with the *SOCS2* mutation. Indeed, 69% of the LDHH4, 92% of the LDHH5 and 97% of the LDHH11 heterozygous carriers were C/T for the *SOCS2* mutation but only 16%, 18% and 23% of the heterozygotes for the *SOCS2* mutation carried one of these haplotypes, respectively (see Additional file 5: Table S4). This could be explained by the fact that the *SOCS2* mutation is carried by two different haplotypes, one in strong linkage disequilibrium with LDHH4–5 and the other with LDHH11. Thus, linkage disequilibrium with the *SOCS2* mutation may explain the observed effect of LDHH4–5 and LDHH11 on milk traits. However, OAR3:129722200C>T in *SOCS2* is not a candidate lethal mutation associated with these haplotypes that could explain their deficit of homozygotes. We hypothesize that a recessive lethal allele located in one of those haplotypes is in partial linkage disequilibrium with the mutant allele of *SOCS2*. Maintaining frequencies of LDHH4–5 (4%) and LDHH11 (7%) in the Lacaune population would then not be explained by a heterozygous advantage of deleterious mutations but by genetic hitchhiking due to *SOCS2* and its association with selected dairy traits [5].

## Conclusions

In this study, we demonstrated the possible segregation of recessive lethal alleles in a sheep population by applying a reverse genetic screen approach on a large genotyping dataset and searching for homozygous haplotype deficiency. Among the 11 LDHH genomic regions detected, we hypothesize that at least eight independent recessive mutations cause early embryonic loss, peri/neonatal lethality or severe health defects in young lambs. We identified the most obvious candidate genes that are assumed to be altered by these mutations, and provide strong working hypotheses to identify them by whole-genome sequencing of heterozygous carriers of the different LDHH. Identification of causal mutations, and of the corresponding altered genes, is important to accurately identify the phenotype they control and to improve our knowledge of the fundamental mechanisms underlying the phenotype. As already observed for haplotypes associated with deleterious recessive mutations, particularly in dairy cattle, most of the LDHH evidenced in dairy sheep were associated with decreased fertility, but also had positive pleiotropic effects on milk production. Management of these haplotypes—or of their causal mutations once they are discovered—in the Lacaune dairy sheep selection scheme through reasoned mating of carrier rams and putative carrier ewes could improve overall fertility and lamb viability. Moreover, if these mutations segregate more widely in other ovine populations, the consequences would apply more broadly to sheep breeding in general.

## Supplementary Information

**Additional file 1: Figure S1.** Distribution of genotyped animals. Figure S1 shows the number of genotyped animals according to sex and year of birth.

**Additional file 2: Table S1.** Clustering HHD in LDHH regions. Table S1 shows all significant haplotypes of 20 markers (i.e., 266 HHD with frequency > 1%, P-value < 1.9 × 10^−4^ and deficit ≥ 75%). As described in the “Methods” section, the 266 HHD could be associated with 11 LDHH regions which are shown in green. **Table S2.** SNPs defining the LDHH regions. Table S2 gives the position of each SNP within LDHH regions according to the sheep reference genome v2.0 and v3.1, and the phased alleles of each deficient haplotype.

**Additional file 3: Table S3.** Positional candidate genes within LDHH regions. Table S3 summarizes all the protein coding genes that are located within the 11 LDHH regions extended by 1 Mb on each side. Genomic coordinates refer to the sheep reference genome v3.1. When available, gene information and association with autosomal recessive disorders in mammals are reported for each protein coding gene based on the following databases: NCBI Entrez: https://www.ncbi.nlm.nih.gov/Web/Search/entrezfs.html; MGI: www.informatics.jax.org; IMPC: https://www.mousephenotype.org); OMIM: Online Mendelian Inheritance in Man (https://omim.org) and OMIA: Online Mendelian Inheritance in Animal (https://omia.org).

**Additional file 4: Figure S2.** Manhattan plot of HHD. Each point represents one haplotype of 20 markers with a frequency > 1% in the maternal phase. The red line represents the P-value threshold (1.9 × 10^−4^) to consider a haplotype significantly deficient in homozygotes. Only HHD with a deficit in homozygotes ≥ 75% were selected and resulted in the identification of 266 significant HHD (represented by green dots).

**Additional file 5: Table S4.** Contingency table between LDHH4, 5 and 11 status and genotypes for the *SOCS2* mutation OAR3:129722200C>T. Ram genotypes at both *SOCS2* OAR3:129722200C>T and LDHH loci.

## Data Availability

The datasets analyzed during this study are available from the corresponding author on reasonable request.
