# Supplementary figures and images for: Identification of homozygous haplotypes carrying putative recessive lethal mutations that compromise fertility traits in French Lacaune dairy sheep
Source: Genet Sel Evol. 2021 May 1;53:41. doi: 10.1186/s12711-021-00634-1 (PMC8088666; doi:10.1186/s12711-021-00634-1)

**Additional file 1: Figure S1. Distribution of genotyped animals.**

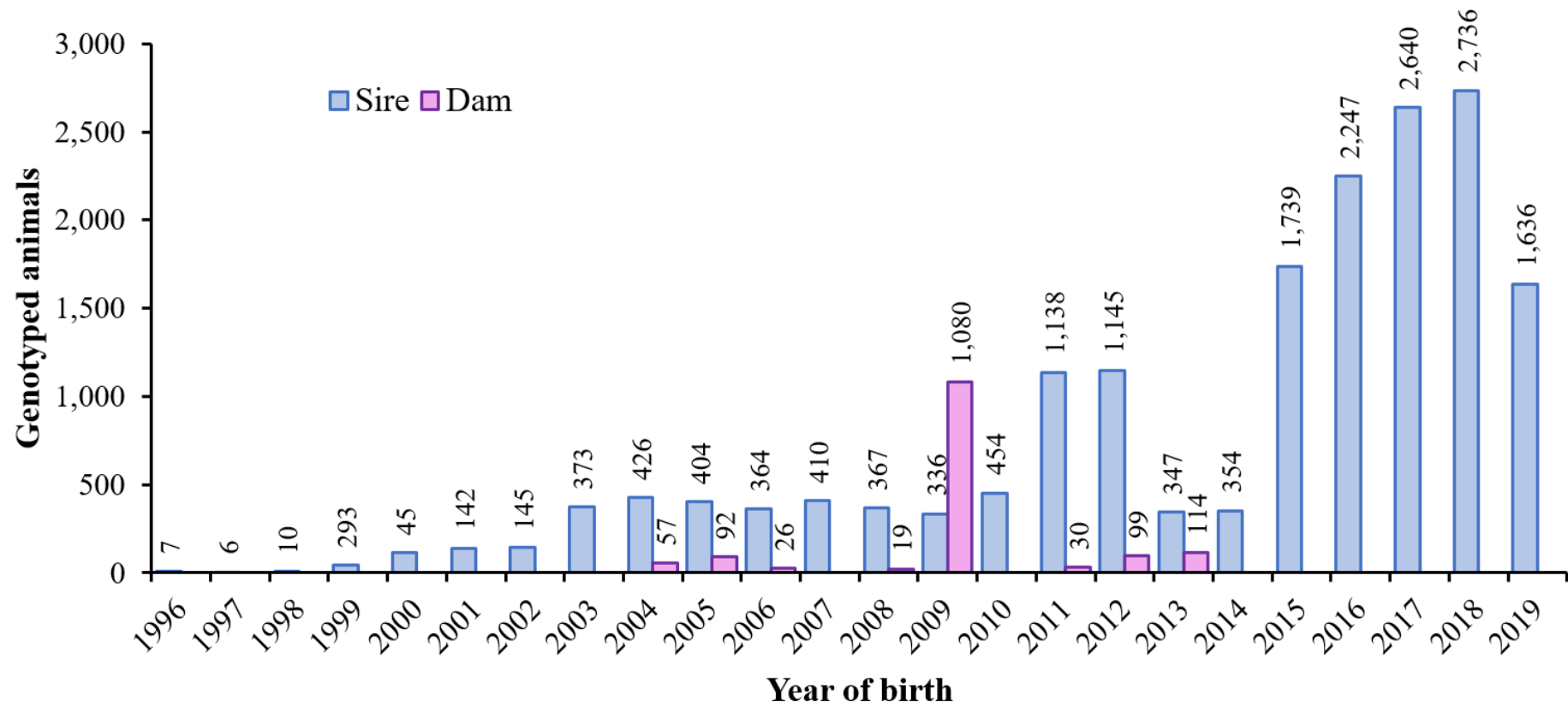

Supplement: Supplementary file 1 — Additional file 1: Figure S1. Distribution of genotyped animals. Figure S1 shows the number of genotyped animals according to sex and year of birth. [file 12711_2021_634_MOESM1_ESM.pdf]

Additional file 4: Figure S2. Manhattan plot of HHD

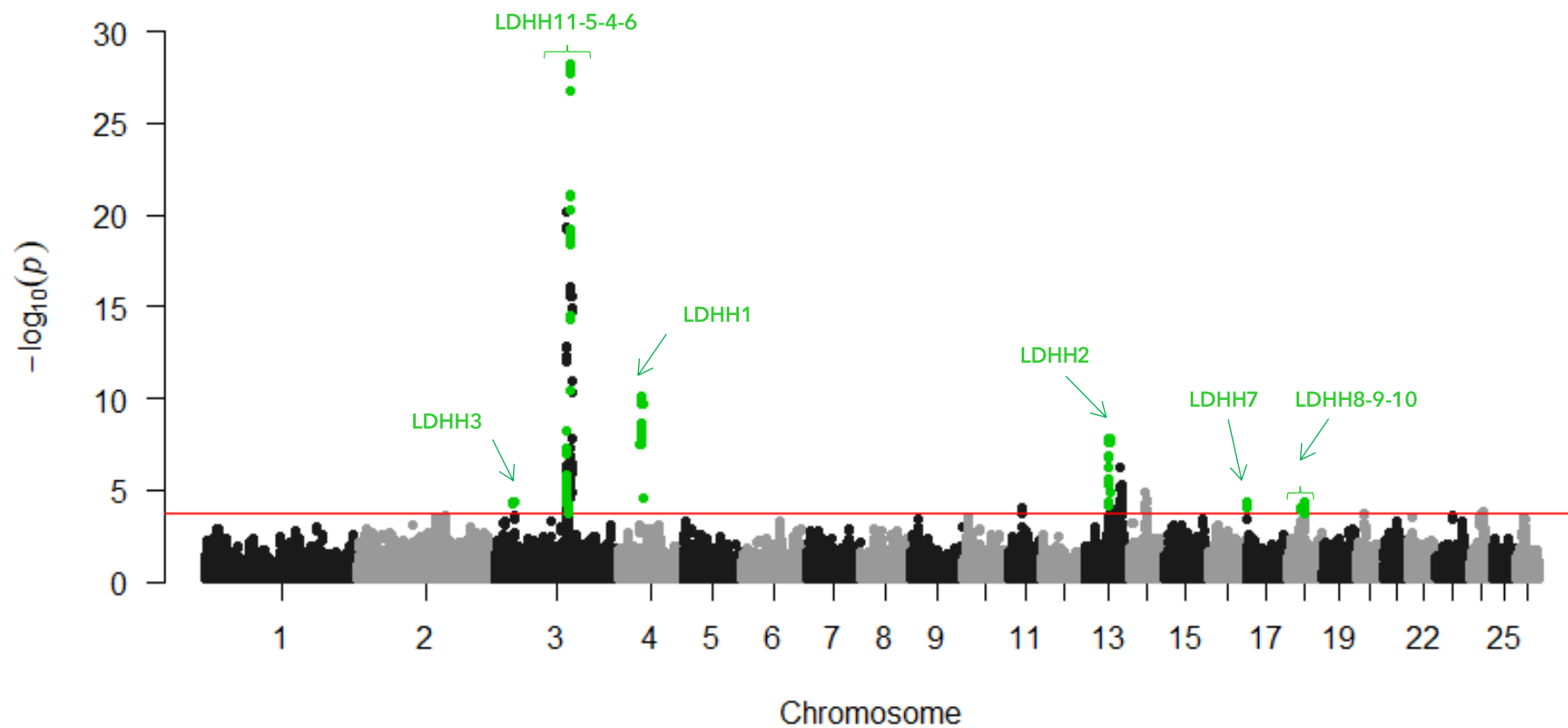

Supplement: Supplementary file 4 — Additional file 4: Figure S2. Manhattan plot of HHD. Each point represents one haplotype of 20 markers with a frequency > 1% in the maternal phase. The red line represents the P-value threshold (1.9 × 10^−4^) to consider a haplotype significantly deficient in homozygotes. Only HHD with a deficit in homozygotes ≥ 75% were selected and resulted in the identification of 266 significant HHD (represented by green dots). [file 12711_2021_634_MOESM4_ESM.pdf]
