# Supplementary material for: Identification of homozygous haplotypes carrying putative recessive lethal mutations that compromise fertility traits in French Lacaune dairy sheep
Source: Genet Sel Evol. 2021 May 1;53:41. doi: 10.1186/s12711-021-00634-1 (PMC8088666; doi:10.1186/s12711-021-00634-1)
Supplement: Supplementary file 5 — Additional file 5: Table S4. Contingency table between LDHH4, 5 and 11 status and genotypes for the SOCS2 mutation OAR3:129722200C>T. Ram genotypes at both SOCS2 OAR3:129722200C>T and LDHH loci. [file 12711_2021_634_MOESM5_ESM.docx]

**Additional file 5: Table S4. Contingency table between LDHH4, -5 and 11 status and genotypes for the *SOCS2* mutation OAR3:129722200C>T.**

|  |  | *SOCS2* genotype (n=) | | |  |
| --- | --- | --- | --- | --- | --- |
|  |  | C/C | C/T | T/T | Total |
| Haplotype | status^a^ | 5,694 | 1,289 | 20 | 7,003 |
| LDHH4 | m/m | 0 | 0 | 0 | 0 |
|  | m/+ | 85 | 206 | 9 | 300 |
|  | +/+ | 5,609 | 1,083 | 11 | 6,703 |
| LDHH5 | m/m | 0 | 0 | 0 | 0 |
|  | m/+ | 11 | 226 | 9 | 246 |
|  | +/+ | 5,683 | 1,063 | 11 | 6,757 |
| LDHH11 | m/m | 0 | 0 | 1 | 1 |
|  | m/+ | 1 | 295 | 7 | 303 |
|  | +/+ | 5,693 | 994 | 12 | 6,699 |

^a^Haplotype status: +/+, non-carrier; m/+, heterozygous; m/m, homozygous carrier
